# Metastatic colon adenocarcinoma to the gingiva treated with spatial fractionation radiotherapy: a case report

**DOI:** 10.3389/fonc.2025.1580430

**Published:** 2025-08-04

**Authors:** Xinyi Liu, Ke Xu, Quan Yao, Jian Fang, Weiyi Zhou, Jinyi Lang

**Affiliations:** ^1^ Department of Radiation Oncology, Sichuan Clinical Research Center for Cancer, Sichuan Cancer Hospital & Institute, Sichuan Cancer Center, School of Medicine, University of Electronic Science and Technology of China, Chengdu, China; ^2^ Department of Radiation Oncology, Radiation Key Laboratory of Sichuan Province, Sichuan Clinical Research Center for Cancer, Sichuan Cancer Hospital & Institute, Sichuan Cancer Center, University of Electronic Science and Technology of China, Chengdu, China

**Keywords:** colonic neoplasms, neoplasm metastasis, radiotherapy, spatially fractionated radiation therapy, gingival metastasis

## Abstract

Metastatic colon adenocarcinoma to the gingiva is exceedingly rare, accounting for 1–3% of maxillofacial malignancies and usually associated with advanced disease and poor prognosis. This case report describes a 68-year-old woman diagnosed with metastatic colon adenocarcinoma presenting with a metastatic lesion measuring 5 × 3 cm located in the left maxilla. The patient received hybrid spatial fractionation radiotherapy (SFRT) combining stereotactic body radiotherapy (SBRT) with conventional SFRT protocols. Post-radiotherapy, the lesion regressed significantly, bleeding ceased, and oral function improved. However, the patient passed away less than two months after the radiotherapy, due to the high systemic tumor burden and severe disease progression. This case highlights SFRT’s efficacy in local symptom control for rare gingival metastases, despite systemic progression. This report underscores the need for optimized SFRT protocols and multidisciplinary approaches in managing metastatic colorectal cancer.

## 1 Introduction

Metastatic colon adenocarcinoma to the gingiva is a rare occurrence, as oral cavity metastases from primary cancers are uncommon, constituting merely 1% to 3% of all malignancies within the maxillofacial region (1). Mandibular metastases are most common (66%), while gingival metastases dominate soft tissue sites (54%) (2–4). Primary tumors often originate from the breast, lung, stomach, colon, or kidney (3–5).

This case demonstrates the innovative application of hybrid spatially fractionated radiotherapy (SFRT) for gingival metastasis. This approach redefines palliative care for oral metastases, achieving a 70% volume reduction with only Grade 2 toxicity, and highlights SFRT’s potential for complex, treatment-resistant lesions.

## 2 Case presentation

**Figure 1:**
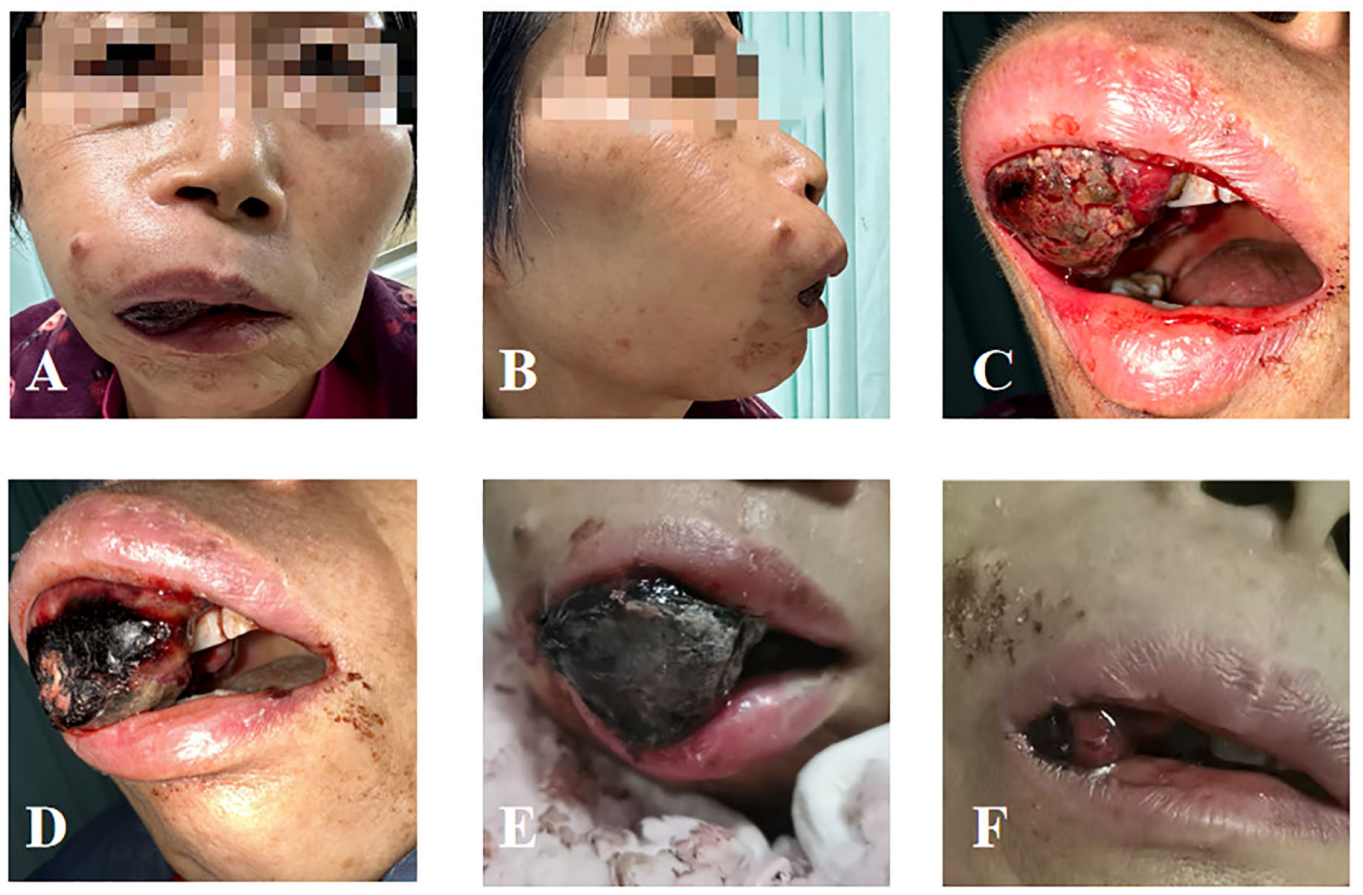
Local condition of the patient’s oral metastasis. **(A)** Anterior view before radiotherapy. **(B)** Lateral view before radiotherapy. **(C)** Intraoral condition before radiotherapy. **(D)** Intraoral condition on the day of completion of radiotherapy. **(E)** One week after radiotherapy. **(F)** Half a month after radiotherapy.

A 68-year-old woman presented with fatigue in June 2023. She then sought medical treatment at the Sichuan Cancer Hospital in Chengdu, Sichuan Province, China, where colonoscopy revealed an ulcerative lesion 60 cm from the anal margin, confirmed as transverse colon adenocarcinoma by biopsy. Following five cycles of FOLFOX chemotherapy from July to September 2023, she underwent laparoscopic right hemicolectomy in October 2023, resulting in a pathological staging of ypT3N2Mx. Postoperative chemotherapy (cycles 6–11) and bevacizumab therapy were administered from November 2023 to January 2024, although the latter was halted due to infusion-related chest discomfort. Subsequent imaging from May to September 2024 indicated progressive lymphadenopathy in the neck and mediastinum, leading to treatment with intensity-modulated radiotherapy (IMRT) at a dose of 40 Gy over 20 fractions, followed by an additional 10 Gy in 5 fractions. Oral capecitabine (1 g twice daily, days 1–14, every 3 weeks) was initiated in September 2024. However, in November 2024, the patient began experiencing gingival pain and bleeding, with the oral mass continuing to bleed and enlarge. Specialized examination revealed that the right side of the maxillofacial region was markedly elevated, with no obvious skin abnormalities. Intraoral examination revealed a soft tissue mass, approximately 5 × 3 cm in size, encircling all teeth in the right maxilla and the central incisor in the left maxilla. The mass had a cauliflower-like surface, with areas of necrosis and active bleeding. It had a foul odor, soft texture, obvious tenderness, and poor mobility. It encircled part of the permanent teeth (**Figures 1A–C**).

**Figure 2:**
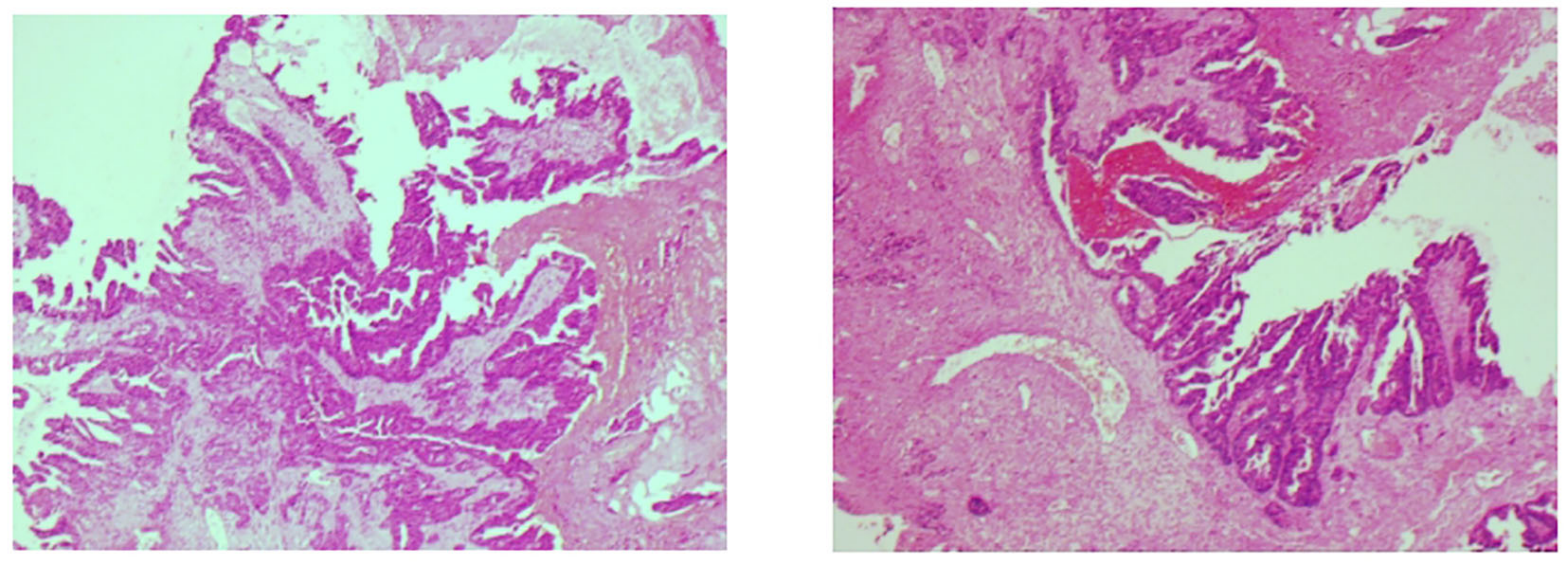
Histopathological examination of the gingival (Hematoxylin-Eosinstaining 20×).

The colorectal tumor marker analysis revealed a carcinoembryonic antigen level of 9.67 ng/ml. Enhanced MRI scans of the skull and oral cavity showed a soft tissue mass in the upper right gingival area, raising concerns about possible metastasis. Additionally, multiple nodules were identified in both hemispheres of the brain, suggesting brain metastasis, along with abnormal signal intensity in the left frontal bone, which could indicate osteoma or other lesions. Whole-body imaging via FDG PET/CT revealed multiple intracranial nodules, the right gingival mass, and several lymph nodes in both clavicles and the mediastinum, as well as multiple nodules in both lungs, all exhibiting varying degrees of metabolic activity, indicative of tumor metastasis. Immunohistochemistry of gingival specimens showed CK20(+), CDX-2(+), SATB2(+), and Ki67(90%+), consistent with a lower gastrointestinal origin (**Figure 2**).

In January 2025, the patient underwent spatially fractionated radiotherapy (SFRT) for a gingival lesion using a hybrid protocol. The treatment planning was performed using the Monaco treatment planning system, with meticulous delineation of the target volumes and optimization of the radiation dose distribution. Subsequently, the treatment plan was rigorously evaluated by both the physician and the medical physicist to ensure its accuracy and safety.

**Figure 3:**
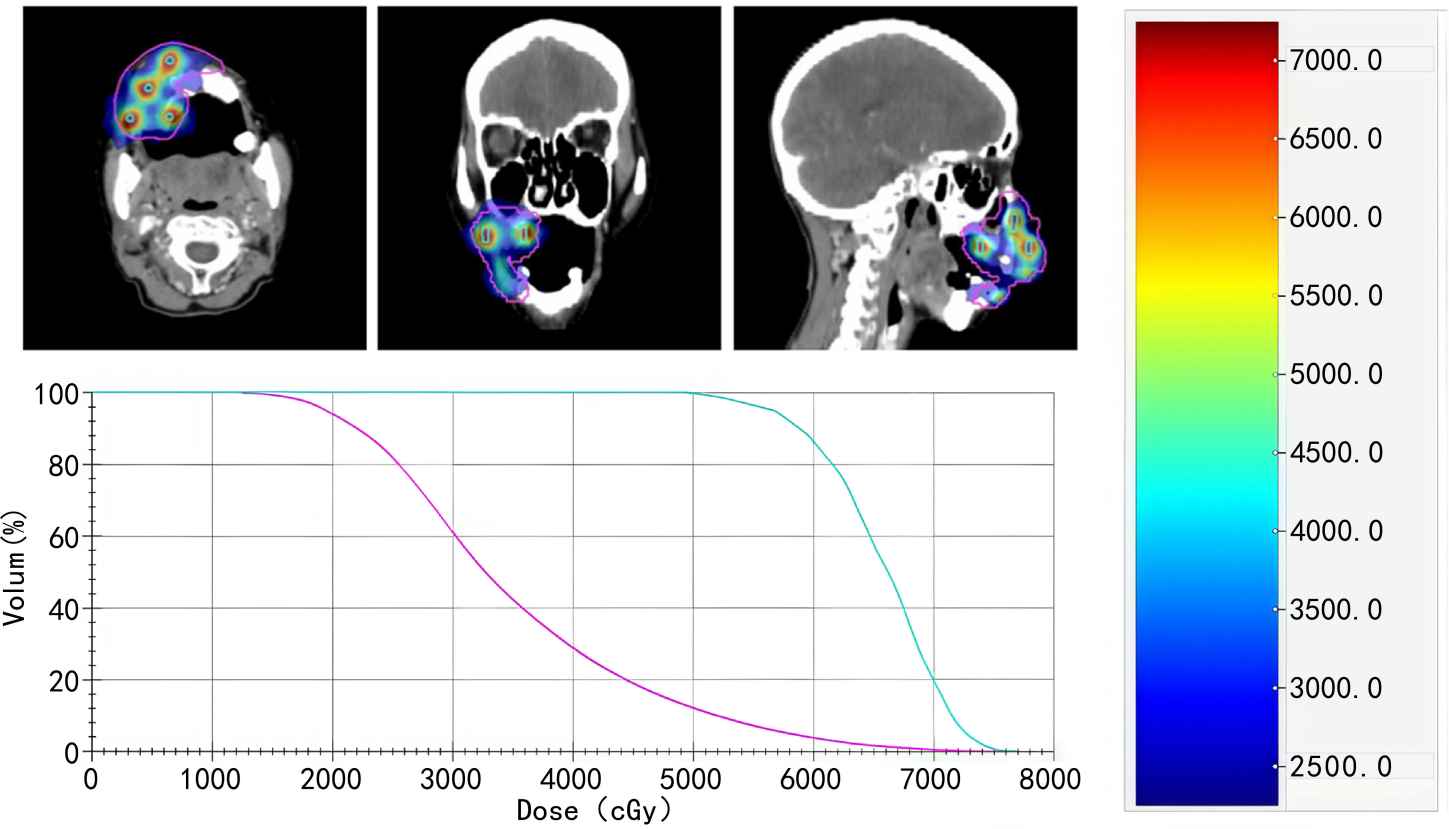
The target volumes and dose distribution of the SFRT plan, as well as the dose-volume histograms (DVHs) for GTV and GTV-H).

The gross tumor volume (GTV) was defined to encompass the entire macroscopic tumor. Spherical vertices (Ø0.4 cm) within GTV receiving peak doses were named GTV-H, prioritizing placing the peak points in the high metabolic areas on PET-CT. Given the unique anatomical location of the patient’s tumor, the spacing between each pair of spheres was carefully controlled to be as close to 2 cm as possible, ensuring uniform distribution of these spheres within the GTV.GTV1 was defined as the potential tumor-infiltrating region located at the upper part of the GTV, near the infraorbital area. GTV2 was delineated as the potential tumor-infiltrating region surrounding the alveolar bone on the lower medial side of the GTV. This target volume segmentation was performed to protect the adjacent organs at risk, achieve a more favorable and safer dose gradient, and enhance the tumor cell kill in potential tumor-involved areas. The treatment plan was implemented using an Elekta Infinity 6 MV linear accelerator. The radiotherapy plan consisted of two components: (1) Stereotactic body radiotherapy (SBRT): GTV received 400 cGy per fraction, twice daily; GTV1 received 300 cGy per fraction, twice daily; GTV2 received 250 cGy per fraction, twice daily. (2) SFRT: GTV received 250 cGy per fraction, twice daily; GTV-H received 750 cGy per fraction, twice daily. The two radiotherapy plans were each implemented for one day (i.e., each plan involved a total of 2 fractions) (**Supplementary Table S1** shows the dose prescription for the SBRT and SFRT components). Efforts were made to achieve ≥80% prescription dose coverage for at least 80% of PTV 250 and ≥85% prescription dose coverage for at least 85% of PTV 750. **Figure 3** illustrates the target volumes and dose distribution of the SFRT plan, as well as the dose-volume histograms (DVHs) for GTV and GTV-H. Organ-at-risk constraints adhered to the American Association of Physicists in Medicine (AAPM) Task Group 101 guidelines for 1 fraction SBRT. Specific TG101 constraints are as follows: Spinal cord: Dmax *<* 14 Gy; Mandible: V30 *<* 30%; Oral mucosa: Dmean *<* 20 Gy; Optic nerve: Dmax *<* 12 Gy; Pituitary gland: Dmax *<* 50 Gy; Brainstem: Dmax *<* 15 Gy.

A head-neck-shoulder mask with oral bite-block ensured immobilization. Cone-beam CT (CBCT) was performed before each treatment to verify tumor localization. The monitor units for each treatment session were about 373.

After radiotherapy, the gingival mass significantly regressed, and bleeding stopped (**Figure 1D**). One week after the completion of the first phase of treatment, the patient’s oral tumor ulcer had crusted over. The volume of the metastatic lesion in the upper jaw had decreased compared to before, and the gingival lesion had protruded out of the oral cavity (**Figure 1E**). Half a month later, a large volume of the tumor within the oral cavity had completely detached. The patient’s local symptoms were significantly alleviated (**Figure 1F**), and restored oral intake. After radiotherapy, the patient developed acute toxicity of Radiation Therapy Oncology Group (RTOG) grade 2.Following the initial phase of radiotherapy, the patient received a single dose of dual immunotherapy with Epalrestat and Toreliumab. However, due to poor overall condition and severe disease progression, the patient decided to forgo further treatment. The timeline of the patient’s key clinical data is presented below (**Table 1**).

**Table 1:** The timeline of the patient’s key clinical data.

Timepoint	Diagnostic/Therapeutic Event	Key Findings/Parameters
Jun 2023	Colonoscopy & biopsy	Ulcerative lesion 60 cm from anal margin; Transverse colon adenocarcinoma
Jul-Sep 2023	Neo-adjuvant chemotherapy (Cycles 1-5)	FOLFOX regimen
Oct 2023	Laparoscopic right hemicolectomy	Pathological staging: ypT3N2Mx
Nov 2023-Jan 2024	Adjuvant therapy	FOLFOX (Cycles 6-11) + Bevacizumab* (*discontinued due to infusion reaction)
May-Sep 2024	Surveillance imaging	Progressive cervical/mediastinal lymphadenopathy
Aug-Sep 2024	Radiotherapy	IMRT: 40 Gy/20 fx, Boost: 10 Gy/5 fx
Nov 2024	Symptom; Laboratory/Imaging; Histopathology	Disseminated systemic metastases with prominent local symptoms in gingival region
Jan 2025	Hybrid SFRT/SBRT	SBRT: GTV(4Gy)/GTV1(3Gy)/CTV(12.5Gy) BID; SFRT: GTV/GTV1(2.5Gy)/H-GTV(8Gy) BID
After Radiotherapy	Immunotherapy	Epalrestat + Toripalimab ×1 cycle
Feb 2025	Treatment cessation	Tumor detachment; oral intake restored; The patient passed away due to multiple metastases

## 3 Discussion

Oral cavity metastases are rare (1–3% of maxillofacial malignancies), typically occurring in molar/premolar regions and conferring poor prognosis (1, 6). Colorectal metastases to the oral cavity are exceptionally uncommon, with 40 reported cases (1916–2024): 28 jawbone and 12 mucosal (3). Median latency from primary diagnosis to gingival metastasis is 9.7 months, with post-metastasis survival of 5.2 months (7). Given the rarity of gingival metastasis, timely diagnosis presents a significant challenge for both clinicians and pathologists.

Studies have demonstrated that the CK7(–)/CK20(+)/CDX-2(+) immunophenotype typically characterizes gastrointestinal metastases, with CDX-2(+) specifically indicating colorectal origin (8, 9). Combined with the patient’s rectal adenocarcinoma history, immunohistochemistry definitively localized the primary tumor.

Tumor dissemination occurs via direct extension, lymphatic, hematogenous, or implantation routes (10, 11). Synchronous cervical lymph node involvement frequently accompanies gingival metastasis due to interconnected regional lymphatic pathways NN and YG (12). In this case, metastasis progressed anatomically from mediastinal to cervical lymph nodes, culminating in gingival and intracranial lesions. This pattern aligns with Batson’s valveless vertebral plexus mechanism, where intrathoracic pressure redirects flow to head/neck vasculature OV (13), and with portal-systemic circulation seeding vascularized sites like gingiva (14). Additionally, chronic gingival inflammation may further promote metastatic adhesion and colonization via microenvironmental alterations YG (15, 16). This is likely because chronic inflammation is associated with various steps in tumorigenesis, including cellular transformation, promotion, survival, proliferation, invasion, angiogenesis, and metastasis.

Currently, the treatment of gingival metastasis primarily includes medical therapy, surgical intervention, or radiotherapy, with the aim of alleviating local symptoms and improving quality of life (17). In the present case, the decision to forgo initial surgical excision followed by postoperative radiotherapy was based on the patient’s advanced metastatic burden, poor performance status, and the high risk of morbidity associated with aggressive surgery. This aligns with recent findings by Yankov et al., who reported a case of mandibular gingival metastasis treated solely with surgery; however, the authors cautioned that surgical intervention is only feasible in select cases with localized disease and minimal comorbidities (18). While the prognosis for patients with gingival metastasis is typically poor, timely and appropriate treatments can significantly enhance clinical outcomes. Recent evidence suggests that survival in such patients may extend up to 9 months after accurate diagnosis when multidisciplinary therapies are employed (18). 

SFRT has emerged as a prominent subject of interest in the field of radiation oncology. This technique subdivides the treatment area into multiple sub-volumes and alternately applies high and low doses of radiation. The hybrid ultra-hypofractionated radiotherapy protocol employed herein—combining spatially fractionated radiotherapy (SFRT) with stereotactic body radiotherapy (SBRT)—demonstrates distinct therapeutic advantages over conventional palliative regimens for oral cavity metastases. This approach synergizes two radiobiological advantages. For SFRT, the core principle is to non-uniformly distribute high-dose radiation within the tumor volume. This pattern is believed to induce complex biological effects, such as vascular effects, bystander effects, and abscopal effect. It offers distinct advantages for bulky tumors, achieving superior local control rates (80-90%) compared to conventional radiotherapy by intentionally creating an inhomogeneous intra-tumoral dose distribution (19). This approach generates a “peak-to-valley” dose effect, where discrete high-dose regions deliver ablative doses while significantly sparing surrounding normal tissues from excessive radiation damage, particularly crucial for tumors in anatomically challenging locations (20–22). Furthermore, SFRT often utilizes an ultra-hypofractionated regimen, enabling rapid therapeutic achievement (19, 22). The enhanced efficacy is attributed to overcoming radioresistance and inducing beneficial biological effects like vascular modulation and immune activation, while the spatial fractionation inherently reduces normal tissue toxicity (20–22). When designing the SFRT plan, the conventional spherical diameter of the peak area is usually designed to be 1.0 - 2.0 cm, with a spacing of 1.5 - 3.0 cm. Considering that the tumor volume of this patient is relatively small and the location is special, an aspherical arrangement of SFRT irradiation with a diameter of 4 mm and a spacing of 2 cm was implemented, referring to the study by Xu et al. (23). The 2 cm inter-sphere spacing was designed to achieve peak-to-valley dose ratios (PTVDR) *>*5:1, known to enhance bystander effects in radioresistant tumors (22). For SBRT, this component delivers tumoricidal doses (EQD2*>*16 Gy) to the entire GTV, overcoming hypoxia-related radioresistance Anon (24).Whereas established approaches prioritize symptom control through moderate fractionation. For instance, Mochizuki et al.? administered palliative radiotherapy with a fractionated dose of 3 Gy to a group of patients with lingual metastasis, reaching a total dose of 30 Gy. Wu et al. (17) reported a case of a patient with gastric cancer metastasis to the gingiva, who received 3D–CRT (50Gy/25F/5w), concurrently with sequential chemotherapy. The symptoms in the above-mentioned cases were all alleviated after treatment. However, our regimen delivered ablative biological doses in minimal fractions. Critically, this strategy achieved unprecedented local control velocity: hemostasis within 24 hours, macroscopic tumor detachment by D15, and functional oral recovery—surpassing the 4–6 week response kinetics documented in conventional cohorts (17, 18, 25). This strategy achieved rapid tumor regression, hemostasis, and lesion detachment within two weeks—outcomes aligning with SFRT’s documented efficacy in managing bulky, radioresistant tumors (22, 26, 27). For example, Xu et al. P et al. (23) administered Lattice SFRT to 19 patients with locally advanced, large head and neck tumors. The results showed that 84.2% of the patients achieved objective response, with 10 patients experiencing partial response and 3 patients having tumor volume reduction exceeding 75%. Moreover, the radiation-related toxicities were within an acceptable range. Another study utilized Grid SFRT in combination with conventional fractionated external beam radiotherapy for large head and neck tumors. The results indicated that among the 12 patients receiving palliative treatment, 54.5% experienced symptom improvement; whereas among the 9 patients treated with curative intent, 44.4% achieved clinical complete response (26).

For this case of a patient with advanced large-volume tumor, SFRT also holds significant value as a palliative radiotherapy modality. It can promote rapid tumor shrinkage and has a favorable safety profile. This approach can alleviate local symptoms, improve quality of life, and enhance prognosis. Additionally, the Grade 2 skin and mucosal-related adverse reactions observed in this case further confirm its favorable toxicity profile compared to IMRT-related complications (17), although this may be related to the short survival follow-up period after treatment in this patient.

Mechanistically, SFRT’s heterogeneous dose distribution may potentiate immunomodulatory effects through enhanced antigen presentation and T-cell activation (27–29). Although synergistic effects with immune checkpoint inhibitors (Epalrestat + Toripalimab) remain undetermined due to abbreviated administration, the observed local response underscores SFRT’s capacity to mitigate tumor-mediated immunosuppression. This contrasts with Xu et al.’s lattice SFRT cohort (23), where sustained immunotherapy integration contributed to prolonged response.

## 4 Conclusions

Gingival metastasis from colorectal adenocarcinoma is rare and often indicates a poor prognosis. In this case, the patient underwent a hybrid SFRT protocol combining SBRT, which achieved notable local control. Following treatment, the gingival mass regressed by approximately 70% in volume, bleeding ceased entirely, and oral ulceration crusted within one week. By two weeks post-treatment, the protruding gingival lesion had detached, restoring oral intake and significantly improving quality of life. However, despite this local success, the patient’s advanced systemic disease precluded long-term survival. Although a single dose of dual immunotherapy was administered, treatment discontinuation due to rapid disease progression limited the evaluation of systemic therapy benefits.

This outcome underscores that SFRT demonstrates robust efficacy in palliating locally aggressive oral metastases. What’s more, the interplay between localized radiotherapy and systemic therapies remains underexplored in metastatic settings. Future studies should prioritize dose escalation strategies for SFRT subvolumes while minimizing toxicity, as well as biomarker-driven selection of patients likely to benefit from combined radiotherapy and immune checkpoint inhibition.

## Data Availability

The original contributions presented in the study are included in the article/**Supplementary Material**. Further inquiries can be directed to the corresponding author.
