# Supplementary material for: Metastatic colon adenocarcinoma to the gingiva treated with spatial fractionation radiotherapy: a case report
Source: Front Oncol. 2025 Aug 4;15:1580430. doi: 10.3389/fonc.2025.1580430 (PMC12358431; doi:10.3389/fonc.2025.1580430)
Supplement: Supplementary file 1 [file Table1.docx]

| Volume | Dose/fx | Fractions | Total |
| --- | --- | --- | --- |
| GTV-H（SFRT） | 7.5Gy | 2 | 15Gy |
| GTV（SFRT） | 2.5Gy | 2 | 5Gy |
| GTV（SBRT） | 4Gy | 2 | 8Gy |
| GTV1（SBRT） | 3Gy | 2 | 6Gy |
| GTV2（SBRT） | 2.5Gy | 2 | 5Gy |

**Supplementary Table S1 The dose prescription for the SBRT and SFRT components**
